# Characterization and Validation of In Vitro and In Vivo Models to Investigate TNF-α-Induced Inflammation in Retinal Diseases

**DOI:** 10.1167/tvst.11.5.18

**Published:** 2022-05-17

**Authors:** Carina M. Weigelt, Nina Zippel, Holger Fuchs, Anna-Kaisa Rimpelä, Tanja Schönberger, Birgit Stierstorfer, Remko A. Bakker, Norbert H. Redemann

**Affiliations:** 1 Cardiometabolic Diseases Research, Boehringer Ingelheim Pharma GmbH & Co. KG, Biberach, Germany; 2 Drug Discovery Sciences, Boehringer Ingelheim Pharma GmbH & Co. KG, Biberach, Germany; 3 Nonclinical Drug Safety, Boehringer Ingelheim Pharma GmbH & Co.KG, Biberach, Germany

**Keywords:** tumor necrosis factor alpha, TNF-α, retinal inflammation, vasculitis, retinopathy, uveitis, AAV, golimumab, adhesion, mouse model, epiretinal membrane

## Abstract

**Purpose:**

Inflammation is implicated in the etiology of diverse retinopathies including uveitis, age-related macular degeneration or diabetic retinopathy. Tumor necrosis factor alpha (TNF-α) is a well-known proinflammatory cytokine that is described as a biomarker for inflammation in diverse retinopathies and therefore emerged as an interesting target to treat inflammation in the eye by neutralizing anti-TNF-α antibodies.

**Methods:**

Recently, we have demonstrated that Adeno-associated virus (AAV)–mediated expression of human TNF-α in the murine eye induces retinal inflammation including vasculitis and fibrosis, thereby mimicking human disease-relevant pathologies. In a proof-of-mechanism study, we now tested whether AAV-TNF-α induced pathologies can be reversed by neutralizing TNF-α antibody treatment.

**Results:**

Strikingly, a single intravitreal injection of the TNF-α antibody golimumab reduced AAV-TNF-α–induced retinal inflammation and retinal thickening. Furthermore, AAV-TNF-α–mediated impaired retinal function was partially rescued by golimumab as revealed by electroretinography recordings. Finally, to study TNF-α-induced vasculitis in human in vitro cell culture assays, we established a monocyte-to-endothelium adhesion co-culture system. Indeed, also in vitro TNF-α induced monocyte adhesion to human retinal endothelial cells, which was prevented by golimumab.

**Conclusions:**

Overall, our study describes valuable in vitro and in vivo approaches to study the function of TNF-α in retinal inflammation and demonstrated a preclinical proof-of-mechanism treatment with golimumab.

**Translational Relevance:**

The AAV-based model expressing human TNF-α allows us to investigate TNF-α–driven pathologies supporting research in mechanisms of retinal inflammation.

## Introduction

Tumor necrosis factor alpha (TNF-α) is a well-characterized proinflammatory cytokine that plays a central role in several diseases with underlying inflammation such as arthritis and inflammatory bowel diseases.^1^ Therefore neutralizing anti-TNF-α biotherapeutics such as golimumab (Simponi), infliximab (Remicade), adalimumab (Humira), certolizumab (Cimzia), or etanercept (Enbrel) are popular steroid-sparing agents in the treatment of TNF-α–mediated inflammation in many rheumatologic disorders, including chronic plaque psoriasis, juvenile idiopathic arthritis, ulcerative colitis, and Crohn's disease for many years.^2^^,^^3^ Elevated TNF-α levels^4^^,^^5^ have also been demonstrated to play a mechanistic role in uveitis, a vision-threatening disease characterized by severe intraocular inflammatory processes. Next to the acute uveitis symptoms including red and painful eyes, floaters, and depressed visual acuity, an uncontrolled uveitis also puts patients at risk for long-term complications, such as formation of cataract, cystoid macular edema, epiretinal membranes, or posterior synechiae.^6^^,^^7^ Since 2016, adalimumab has been approved by the FDA for systemic treatment of noninfectious intermediate, posterior, and pan-uveitis based on the results of phase 3 trials VISUAL I and VISUAL II.^8^^,^^9^ Beyond the acute inflammation in uveitis, chronic inflammatory processes came also into focus for other retinopathies such as proliferative diabetic retinopathy (DR) and age-related macular degeneration (AMD).^10^^–^^12^ TNF-α is upregulated in the vitreous and aqueous humor, serum and tear fluid of proliferative DR patients^13^^–^^16^ and case reports have shown an improvement in visual acuity in AMD patients after infliximab treatment.^17^

Single-cell sequencing studies of human retina or retinal pigment epithelium (RPE) tissue have detected expression of TNF-α restricted to microglia or infiltrated immune cells.^18^^,^^19^ TNF-α is one of the cytokines demonstrated to induce microglial activation in vitro*.*^20^ A vicious cycle, as microglia in their activated state enhance their expression of proinflammatory cytokines, therefore, further boosting TNF-α levels that may exert multiple functions on retinal cell types. In porcine explant cultures, TNF-α has been shown to induce reactive gliosis of Müller glia with hypertrophy, tissue remodeling and stiffening, which can be prevented with adalimumab.^21^ TNF-α promotes retinal leukostasis, partly by upregulating the expression of adhesion factors, such as ICAM1, E-Selectin and VCAM-1, which are involved in tethering and firm adhesion of leukocytes to the endothelial cells lining the vasculature, but also by stimulating their expression of cytokines, such as CCL2 or IL-6.^22^ All these effects are reflecting aspects of retinal diseases, such as uveitis, diabetic retinopathy, or AMD.

To further elucidate the function of TNF-α in inflammation-related eye diseases, studies in relevant in vivo models would be warranted. A major hindrance in such efforts is the lack of cross-reactivity of most neutralizing TNF-α antibodies between human and disease model species,^23^ presumably due to the low similarity of human and rodent TNF-α. We and others have recently shown that Adeno-associated virus (AAV)-mediated overexpression of murine or human TNF-α induces inflammation in the murine retina.^24^^,^^25^ In our previous study, intravitreal (IVT) injection of AAV-TNF-α resulted in immune cell infiltration into the vitreous, immune cell activation, vasculitis, and development of fibrotic epiretinal membrane-like structures. In contrast to approaches using IVT injection of recombinant TNF-α,^26^^,^^27^ AAV-induced expression of TNF-α allows for long-term investigations. Similar pathologies have been observed in patients with uveitis and the experimental autoimmune uveitis (EAU) mouse model.^28^^–^^31^ In LPS-induced uveitis in the rabbit, treatment with anti-TNF-α effectively lowered the disease score,^32^ further highlighting the mechanistic role of TNF-α in uveitis. Similarly, in the *rd10* mouse model of retinal degeneration, adalimumab reduced inflammasome activation and microglial activation and slowed down retinal degeneration.^33^ Thus AAV-driven expression of human TNF-α mimics many aspects of human retinal diseases and may be used as a novel mouse model to further understand the pathophysiologic role of chronic TNF-α-related inflammation in the retina.

In this study, we validated our AAV-TNF-α induced, humanized mouse model by using the therapeutic neutralizing TNF-α antibody golimumab (Simponi) as a proof-of-mechanism treatment. Strikingly, a single IVT treatment with golimumab significantly reduced retinal inflammation and rescued the TNF-α-induced increase in retinal thickness. We further evaluated electroretinography (ERG) recordings as a quantifiable readout to measure treatment efficacy and demonstrated improved photoreceptor function after golimumab treatment. Finally, we established a human monocyte adhesion in vitro model that may be used to transfer results from our mouse model to a human setting.

## Material and Methods

### AAV Production

Cloning and production of AAV-TNF-α and the negative control AAV-stuffer (includes a fragment of the 3ʹ untranslated region of the *UBE3A* gene) has been described elsewhere.^25^^,^^34^^,^^35^ In brief, human TNF-α is expressed under a ubiquitous CAG promoter and packed into ShH10 capsid that primarily infects Müller glia.^36^ AAVs were stored in AAV buffer (phosphate-buffered saline solution, 1 mM MgCl_2_, 2.5 mM KCl, 10% glycerol, 0.001% Pluronic F-68, pH 7.4).

### Animal Experiments

**Figure 1.**
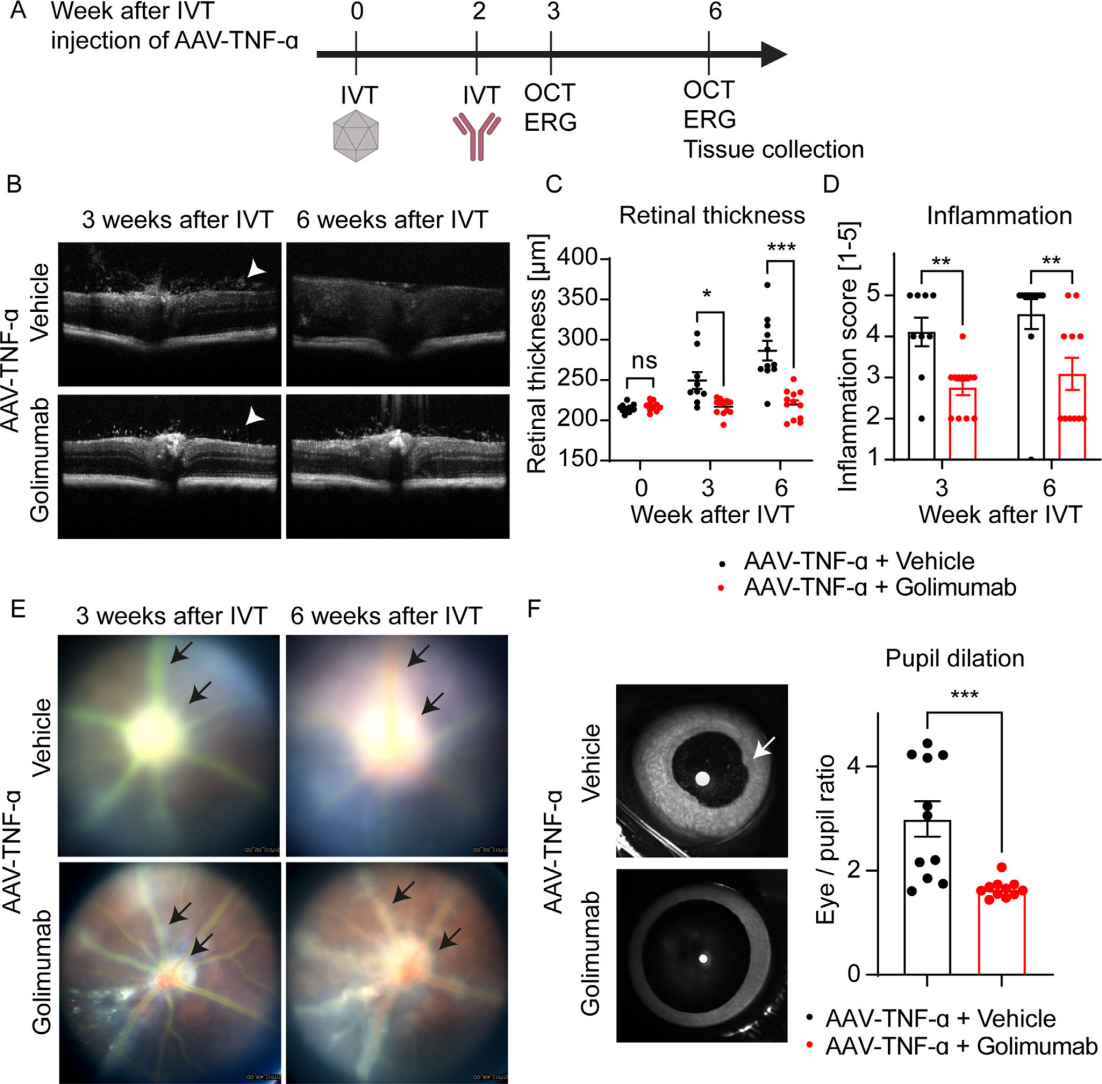
Golimumab improves AAV-TNF-α induced inflammation. (A) Experimental setup: AAV-TNF-α was injected IVT, followed by a subsequent IVT injection of golimumab or vehicle two weeks later. In vivo imaging was done three and six weeks after IVT injection of AAV-TNF-α, and tissues were collected for histological and gene expression analysis at the end of the study. This figure was generated with BioRender.com. (B) OCT scans revealed that AAV-TNF-α induced infiltration of immune cells to the vitreous (*white arrowheads*), and disorganization of the retinal layers was improved by golimumab. (C) Retinal thickness was gradually increased after AAV-TNF-α treatment, which was ameliorated on subsequent golimumab treatment (Mixed-effects analysis: Time: ****P < 0.0001; Treatment: ****P < 0.001, Interaction: ****P < 0.0001; n = 9–12 eyes, Sidak's multiple comparisons test). (D) Inflammation was graded blindly based on an inflammation scoring system and the OCT pictures. Inflammation was significantly reduced in golimumab treated eyes (Mixed-effects analysis: Time: n.s.; Treatment: **P < 0.01, Interaction: n.s.; n = 9–12 eyes, Sidak's multiple comparisons test). (E) Fewer white, cellular infiltrates around the optic nerve and perivascular infiltrates were present in golimumab treated eyes. Note that imaging of severely affected eyes in the AAV-TNF-α group was impaired because pupils were not fully dilated (see F). (F) Pupil dilation was impaired in AAV-TNF-α control group, but not AAV-TNF-α eyes treated with golimumab as quantified by the eye to pupil area ratio (Unpaired *t* test: **P < 0.01, n = 11–12 eyes) six weeks after IVT injection of AAV-TNF-α*.* In all graphs mean ± SEM is shown.

Male and female C57BL/6J mice six to eight weeks old were purchased from Charles River (Sulzfeld, Germany) and housed in individually ventilated cages. Six mice per group (vehicle or golimumab) were used. Ketamine 60 to 90 mg/kg (10%, Medistar Arzneimittelvertrieb GmbH) and 6 to 8 mg/kg xylazine (Rompun; Bayer, Ltd., Marsa, Malta) were injected intraperitoneally to anesthetize the mice for in vivo imaging and ERG analysis (pretreatment value day 0, see Fig. 1A) followed by IVT injection of AAV-TNF-α or AAV-stuffer during the same anesthesia. The second unilateral IVT injection (vehicle or golimumab) was performed two weeks later under short-term inhalation anesthesia with isoflurane. In addition, local anesthetic was applied to the eyes (Novesin, OmniVision Technologies, Santa Clara, CA, USA) before IVT injection. AAVs 1 × 10^9^ VG/eye in 1 µL AAV buffer, 1 µL golimumab (100 mg/mL; Simponi; MSD Sharp & Dohme, Kenilworth, NJ, USA) or double-distilled H_2_O as vehicle control treatment were injected intravitreally with a 34-G needle. Eyes in which the lens or a major blood vessel was injured due to the IVT injection procedure were excluded from analysis. Mice were sacrificed by cervical dislocation, and the eyes were enucleated and snap-frozen in liquid nitrogen or fixed in 4% paraformaldehyde for histological analysis six weeks after injection of AAV-TNF-α or AAV-stuffer. Animal experiments were performed in accordance with the German Animal Welfare Act, the guidelines of the Federation of the European Laboratory Animal Science Association and the ARVO statement for the use of animals in ophthalmic and vision research. Animal experiments performed in this study were reviewed and approved by the local authorities.

### In Vivo Imaging

In brief, mice were anesthetized, and pupils were dilated with 5 mg/mL tropicamide (Mydrum; Bausch + Lomb, Rochester, NY, USA) and phenylephrine (Neosynephrin-POS 10%; Ursapharm, New Delhi, India). Optical coherence tomography (OCT) recordings were done with a Bioptigen Envisu R2210 device (Leica Microsystems, Wetzlar, Germany) equipped with a lens designed for mouse eyes (50° field of view). Fundus pictures were recorded with a Micron IV Fundus camera (Phoenix Research Laboratories, Pleasanton, CA, USA). Pictures of mouse eyes (pupil size) were taken with a Spectralis HRA/OCT device (Heidelberg Engineering, Heidelberg, Germany) approximately 20 minutes after pupil dilation as described above. The same eyes were imaged three or four times: Before AAV injection (pretreatment value) and one week (AAV-stuffer control only) and three and six weeks after AAV injection.

### ERG

ERG recordings were performed before injection of AAV (pretreatment value) and at one (AAV-stuffer control only), three, and six weeks after AAV injection in the same mice. Mice were dark-adapted over-night and anesthetized as described above with ketamine/xylazine. Pupils were dilated with tropicamide and phenylephrine (see above) and Methocel (2%; OmniVision Technologies, Santa Clara, CA, USA) and gold electrodes were applied to both eyes. As a reference, a clip was placed in the mouth corner of the mouse, and grounding was ensured by another clip positioned at the tail root of the mouse. ERGs were recorded using an Espion E3 Electroretinography system (Diagnosys, Lowell, MA, USA) and a Full-Field Ganzfeld stimulator (ColorDome; Diagnosys) with UV LEDs and a Xenon light. ERG signals were recorded with 0.15 Hz low-frequency and 500 Hz high-frequency cutoffs. The following flash intensities were used for scotopic measurements: 0.00001, 0.0001, 0.001, 0.01, 0.1, 1, and 10 cd ∙ s/m^2^. The amplitude heights of b-waves for each ERG flash response were calculated using the MATLAB software (MathWorks), and the results were graphically depicted in GraphPad Prism.

### Inflammation Grade

To quantify the inflammation stage of a mouse eye based on OCT scans, an inflammation grading system was developed: 1 = normal eye; 2 = few (<30) cellular infiltrates in vitreous; 3 = many (>20) cellular infiltrates in vitreous, retinal layers intact; 4 = cellular infiltrates and retinal layers partially disorganized; 5 = retinal layers fully disorganized, regular OCT image recording not possible. Examples on the OCT inflammation grading are shown in Supplementary Figure S1. The OCT pictures were blinded and scored by an independent scientist. The ratio of pupil size to the whole eye was quantified using the area of the pupil and the whole eye measured as the area of an ellipse within FIJI/ImageJ. Retinal thickness was quantified with the Bioptigen Envisu software and FIJI/ImageJ by measuring the thickness of the retina temporal and nasal at 200 to 300 µm distance from the optic nerve. Data are representative of two measurements per retina.

### Histology

Eyes were enucleated and fixed in 4% paraformaldehyde (AR1068; Boster Bio, Pleasanton, CA, USA) for 48 hours at 4°C. Whole eyeballs were dehydrated and infiltrated with paraffin using a tissue processor (Tissue-Tek VIP 6; Sakura Finetek USA, Inc., Torrance, CA, USA) and subsequently embedded into paraffin blocks on a HistoCore Arcadia Embedding Center (Leica Biosystems, Melbourne, Australia). Three-micrometer sections were cut at the level of the optical nerve and hematoxylin and eosin and Masson's Trichrome staining was performed according to standard protocols. Immunofluorescence staining was carried out on the automated Leica Bond platform (Leica Biosystems) using the Opal Multiplex IHC Kit (Akoya Biosciences, Menlo Park, CA, USA). Antibodies used in this study and details about the antigen retrieval are listed in the Table. Nuclei were stained with spectral DAPI (FP1490; Akoya Biosciences) and slides mounted with ProLong Antifade Mounting Medium (P36961; Invitrogen, Carlsbad, CA, USA). Tissues were imaged using an Axio Scan.Z1 slide scanner (magnification ×20; Carl Zeiss Microscopy GmbH, Jena, Germany). Iba1^+^ and F4/80^+^ area was quantified using HALO image analysis software (Indica Labs, Albuquerque, NM, USA).

**Table.** Antibodies Used in This Study

Antigen	Host	Dilution	Antigen Retrieval	Source
Anti-Iba1	Rabbit	1:1000	HIER (95°C/ 20 min, pH 9.0)	019-19741, Wako
Anti-GFAP	Rabbit	1:8000	HIER (95°C/ 20 min, pH 9.0)	ab7260, Abcam
Anti-F4/80	Rabbit	1:200	HIER (95°C/ 20 min, pH 6.0)	70076, Cell Signaling
RPE65	Rabbit	1:2000	HIER (95°C/ 20 min, pH 9.0)	ab231782, Abcam
OPAL polymer anti-rabbit-HRP secondary antibody	—	1:5	—	SKU ARR1001KT, Akoya Biosciences

HIER, heat-induced epitope retrieval.

### MSD Analysis

Mouse eye lysates were generated by homogenization in ice-cold lysis buffer (9803; Cell Signaling Technology, Danvers, MA, USA) supplemented with proteinase inhibitor Pefabloc SC (76307-100MG; Sigma-Aldrich Corp., St. Louis, MO, USA) with metal beads (15987602; Bertin Technologies, Montigny-le-Bretonneux, France) in a tissue homogenizer (Precellys Evolution; Bertin Technologies). The supernatant was carefully collected after centrifugation at >16,000*g* and 4°C for 10 minutes. Total protein concentration was determined using a bovine serum albumin protein assay (Pierce, Appleton, WI, USA), and lysates were diluted to 5 µg/mL total protein. A customized MSD U-Plex assay (Meso Scale Diagnostics, Rockville, MD, USA) was used to measure human TNF-α and mouse IL-1β, IL-6, CCL2 and MMP-9. MSD assay was performed according to the manufacturer's protocol. SECTOR Imager 6000 (Meso Scale Diagnostics) was used for quantification.

### Cell Culture

Primary human retinal microvascular endothelial cells (HRMEC, ACBRI 181; Cell Systems Corporation, Kirkland, WA, USA) were cultivated in Endothelial Cell Growth Medium (C-22010; Promocell, Heidelberg, Germany) on plates precoated with 0.1% gelatin (ES-006-B; Millipore, Burlington, MA, USA). Immortalized human microglia cells (hMC, P10354-IM; Innoprot, Derio, Spain) were cultivated in microglia cell medium (P60116; Innoprot). THP-1 cells (88081201; ECACC) were cultivated in RPMI medium (BE12-702F) supplemented with 1% GlutaMax (35050061; Gibco, Thermo Fisher Scientific, Waltham, MA, USA) and 10% FCS. For the TNF-α–induced expression analysis assays, cells were stimulated with 10 ng/mL recombinant human TNF-α (H8916, Sigma Aldrich Corp.) for 24 hours with or without 58.8 nM golimumab (Simponi, MSD Sharp & Dohme).

### THP-1 Adhesion Assay

HRMEC cells were cultivated until they reached confluence. Cells were stimulated in a starvation medium containing endothelial basal medium with 0.5% bovine serum albumin with and without TNF-α and golimumab for three hours. THP-1 cells were labeled with the fluorescent live-stain BCECF-AM (B3051; Life Technologies, Carlsbad, CA, USA) at 37°C for one hour and 20,000 THP-1 cells were added onto the HRMEC monolayer and incubated for 30 minutes. Nonadherent THP-1 cells were washed off with phosphate-buffered saline solution and cells were fixed in 4% paraformaldehyde. Imaging and subsequent image analysis was done with the Opera Phenix High-Content Screening System and Harmony software (Perkin Elmer, New York, NY, USA).

### RNA Extraction and q-RT-PCR

RNA was extracted using the RNeasy Mini Kit (74106; Qiagen, Hilden, Germany) according to the manufacturer's protocol. cDNA synthesis was done using the High capacity cDNA Archive Kit (4322169; Applied Biosystems, Foster City, CA, USA) and q-RT-PCR was performed with the TaqMan Fast Advanced Master Mix (44444557; Applied Biosystems) on a Quant Studio 6 Real-Time PCR system (Applied Biosystems). Relative expression (fold induction) was calculated using the ΔΔCT method and the POLR2A gene was used as a normalization control. The following Taqman probes were used in this study: POLR2A (Hs00172187_m1), CCL2 (Hs00234140_m1), MMP9 (Hs00957562_m1), IL1B (Hs1555410_m1), IL6 (Hs00174131_m1), ICAM1 (Hs00164932_m1).

### Statistical Analysis

GraphPad Prism (Version 9.0.0) was used for statistical analysis. Statistical tests are indicated in the respective figure legends. Bar plot graphs represent the mean values ± SEM. The GraphPad Prism function “Identify outliers” was used to remove outliers. Significance was determined according to the *P* value: *P < 0.05, **P < 0.01, ***P < 0.001, ****P < 0.0001.

## Results

### A Single IVT Dose of Golimumab Prevents Retinal Thickening And Posterior Synechia

The aim of this proof-of-mechanism study was to test whether the pathologies induced by a chronic AAV-induced expression of human TNF-α may be reversed by a bolus IVT injection of a TNF-α neutralizing antibody. We first injected mice with AAV-TNF-α intravitreally and two weeks later with a single IVT dose of the therapeutic neutralizing TNF-α antibody golimumab (Fig. 1A). First, we used OCT to assess ocular inflammation and retinal thickness three and six weeks after injection with AAV-TNF-α. Similar to our previous study,^25^ AAV-TNF-α treatment led to infiltration of immune cells into the vitreous three weeks after injection (Fig. 1B, white arrowheads) and disorganized retinal layers and thickening of the retina six weeks after IVT application (Fig. 1B). In contrast, AAV-stuffer control treatment did not lead to immune cell infiltration, and retinal thickness was unchanged (Supplementary Figs. S2A, S2B). Quantification of retinal thickness demonstrated that the IVT administration of golimumab significantly reduced the increase of retinal thickness induced by AAV-TNF-α (Fig. 1C). Furthermore, inflammation was significantly reduced by golimumab as judged by an inflammation grading score (Fig. 1D). Similarly, perivascular infiltrates and infiltrates around the optic nerve were observed after AAV-TNF-α injection and reduced by golimumab treatment (Fig. 1E, black arrows), and not observed by AAV-stuffer treatment (Supplementary Fig. S2C). Interestingly, despite pupil dilation with tropicamide and phenylephrine, pupils in the AAV-TNF-α group were not fully dilated and showed irregular borders (Fig. 1F, white arrow), reminding of posterior synechia observed in patients with chronic inflammation/uveitis. Quantification revealed that golimumab treatment significantly reduced the eye to pupil ratio and thus prevented the posterior synechia-like pupil phenotype (Fig. 1F). Taken together, golimumab clearly reduced the inflammation induced by AAV-TNF-α, as judged using in vivo imaging techniques.

### Golimumab Improves Retinal Function as Measured by Electroretinography

**Figure 2.**
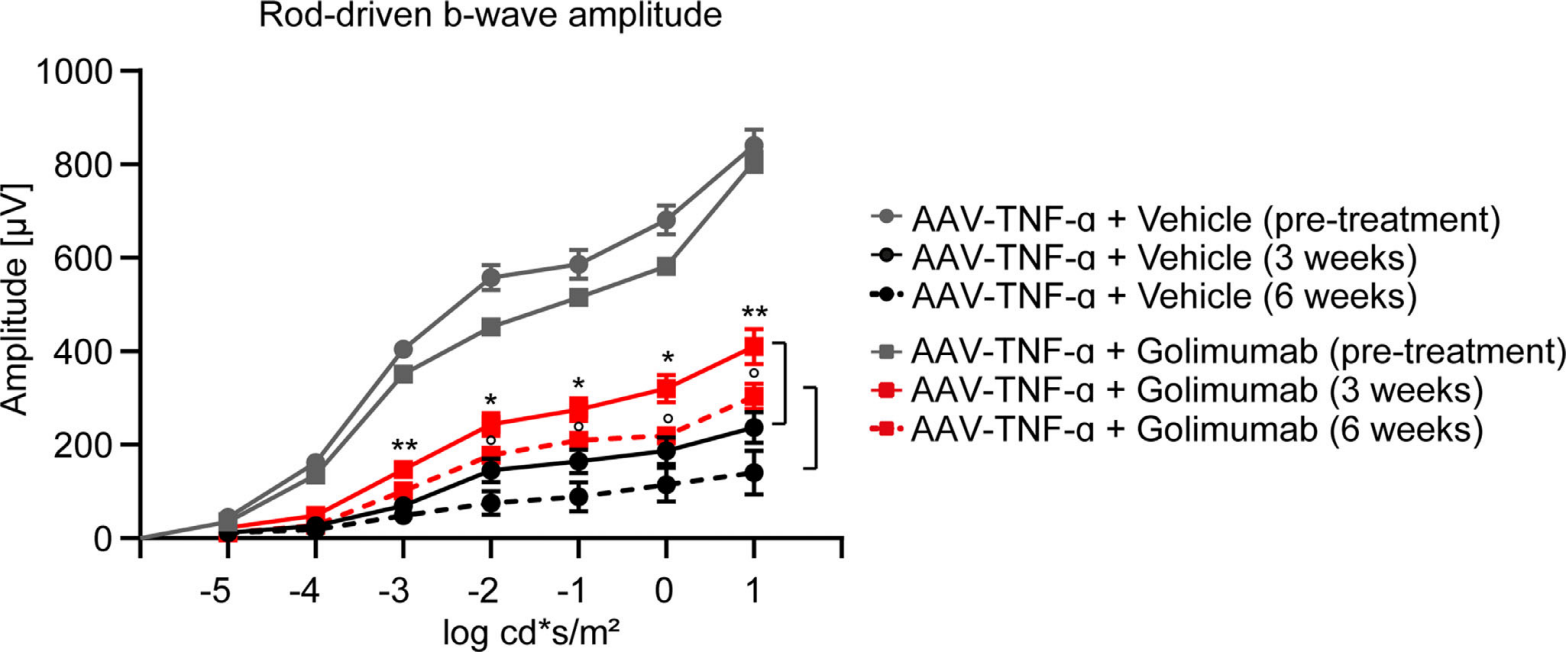
ERG flash responses were improved by golimumab treatment. Rod-driven b-wave amplitudes were reduced by AAV-TNF-α compared to the pretreatment baseline measurement. Golimumab treatment increased the b-wave amplitude compared to the age-matched vehicle control (three weeks vehicle versus three weeks golimumab treatment: *P < 0.05, **P < 0.01; six weeks vehicle versus six weeks golimumab treatment: *P* < 0.05, two-way ANOVA for each flash intensity with Tukey's post-hoc test, n = 11–12, mean ± SEM).

To confirm whether the disorganization of the retinal layers observed by in vivo imaging also impairs retinal function, we used electroretinography (ERG) to analyze and quantify retinal function. Interestingly, AAV-TNF-α induced a decrease in rod-driven b-wave amplitude that was progressing over time compared to the baseline values before IVT injection of AAV-TNF-α (Fig. 2, grey vs. black lines). Remarkably, golimumab treatment significantly increased the b-wave amplitude under scotopic conditions at the 3 and 6 weeks timepoints compared to the age-matched vehicle control (Fig. 2, red lines vs. black age-matched control). However, it is of note that AAV-stuffer control injection alone also significantly reduced ERG response, but much less compared to AAV-TNF-α, potentially due to administration-related effects by the IVT injection, the AAV buffer or the AAV itself (Supplementary Fig. S2D). Altogether, ERG recordings demonstrated a severe impairment of retinal function by AAV-TNF-α, which was partially prevented by a single IVT dose of golimumab.

### Retinal Morphology is Preserved by a Single IVT Dose of Golimumab

**Figure 3.**
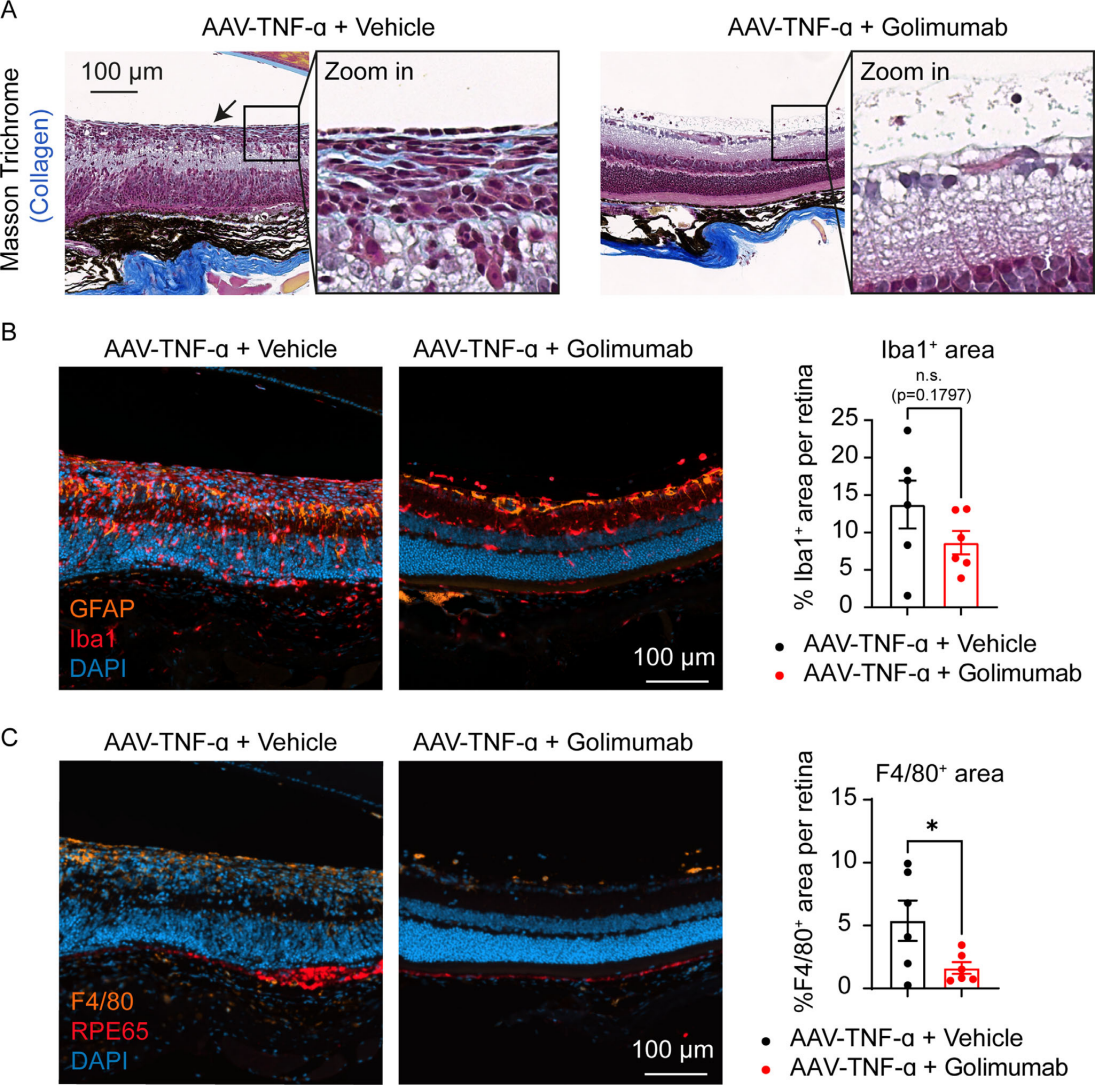
Retinal morphology was preserved by golimumab. (A) Masson's trichrome staining indicated development of an epiretinal-membrane-like, collagen-rich (*blue*) structure on the inner retina of vehicle treated animals (*black arrow*) but not golimumab-treated animals. (B) Immunofluorescent labeling of Iba1^+^ microglia/macrophages (*red*), GFAP^+^ astrocytes/activated Müller glia (*orange*) and nuclei (DAPI, *blue*). Iba1^+^ area was slightly but not significantly reduced in golimumab treated eyes (Unpaired t-test: n.s., mean ± SEM, n = 6 eyes). (C) Immunofluorescent labeling of F4/80^+^ activated microglia/macrophages (*orange*), RPE65^+^ RPE cells (*red*), and nuclei (DAPI, *blue*). F4/80^+^ area (in percent of the total retinal area) was significantly smaller in golimumab treated eyes compared to vehicle treatment in the AAV-TNF-α model (Unpaired *t* test: *P < 0.05, mean ± SEM, n = 6 eyes). Representative pictures were selected based on the average of Iba1^+^ area.

Next, we analyzed histological cross-sections to investigate the treatment effect of golimumab on a cellular level. In line with our previous studies, hematoxylin and eosin staining showed severe pathologies in AAV-TNF-α treated eyes 6 weeks after IVT injection, as seen by disorganized retinal layers, posterior synechia (Supplementary Fig. S3A, black arrowhead) and immune cell infiltrates in the anterior chamber (Supplementary Fig. S3B, C). Overall, retinal morphology was preserved by golimumab treatment, as all eyes had clearly distinguishable retinal layers and only rarely posterior synechia (Supplementary Fig. S3). However, we noticed few immune cells in the vitreous also in the golimumab treatment group, similar to the earlier timepoint 3 weeks after IVT injection of AAV-TNF-α in our previous study,^25^ suggesting delayed but not fully suppressed disease progression by golimumab. In line with our previous data, AAV-TNF-α-treated eyes developed an epiretinal membrane-like fibrotic layer spanning large parts of the inner retina 6 weeks after IVT administration of AAV-TNF-α (Fig. 3A, black arrow). In contrast, in golimumab-treated eyes, epiretinal membranes were absent or reduced to very restricted, small areas around the optic disc (Fig. 3A). Quantification of Iba1^+^ microglia and macrophages revealed a slight, but not significant trend towards less Iba1^+^ cells in golimumab treated eyes (Fig. 3B). In addition, GFAP staining was more localized to the astrocyte layer in golimumab treated eyes suggesting prevention of reactive gliosis in Müller glia (Fig. 3B).

Moreover, we aimed to further characterize the origin of the fibrotic layer in the inner retina identified by Masson Trichrome staining (Fig. 3A). In retinal diseases, several cell types have been identified to differentiate into fibrotic cells by different mechanisms that contribute to disease progression: Reactive gliosis of Müller glia, endothelial-mesenchymal transition, epithelial-mesenchymal transition of RPE cells and macrophage-to-myofibroblast transition (MMT).^37^^–^^39^ Because in most of the cells in the fibrotic layer GFAP staining was absent (Fig. 3B), Müller glia are likely not the main contributor to the development of the fibrotic layer. However, we noticed that the fibrotic cells were Iba1^+^ (Fig. 3B), suggesting that they may originate from macrophages. To strengthen this hypothesis, we used another marker for activated microglia/macrophages (F4/80) and, indeed, found that the fibrotic layer was also F4/80^+^. Quantification of F4/80^+^ cells further revealed that significantly less F4/80^+^ cells were present in golimumab treated eyes, confirming the protective anti-inflammatory effect of golimumab (Fig. 3C). Finally, to test whether epithelial-mesenchymal transition of RPE cells may contribute to the development of the fibrotic layer, we used RPE65 immunostaining to identify RPE cells in the retina. RPE65 staining was present in the RPE layer; however, we did not identify RPE65^+^ cells in the fibrotic layer (Fig. 3C). Notably, the RPE65 staining was more irregular in the vehicle-treated group, as for example seen by areas with a thickened RPE cell layer, suggesting that the RPE is damaged in our AAV-TNF-α model, but it does not contribute to the fibrotic layer in the inner retina. In summary, histopathological analysis confirmed that golimumab partially restored the retinal morphology in AAV-TNF-α–treated animals and that the fibrotic layer likely origins from macrophages.

### Expression of Proinflammatory Cytokines is not Significantly Affected by Golimumab Treatment

Next, since inflammation was seen in both the anterior and posterior part of the eye, we measured proinflammatory cytokines in whole mouse eye lysates. First, we confirmed that human TNF-α expression was induced by AAV-TNF-α treatment (Supplementary Fig. S4, upper left panel). We have previously shown that AAV-TNF-α treatment significantly increased expression of mouse IL-1β, IL-6, CCL2 and MMP-9 compared to animals treated with AAV-stuffer (non-coding) negative control. We observed similar trends in this study compared to the AAV-stuffer control, but only the change in expression of CCL2 was statistically significant (Supplementary Fig. S4). As expected, expression of golimumab slightly reduced the expression of all measured cytokines, however, due to high variation, none of the changes were significant (Supplementary Fig. S4). Future studies with a higher number of animals will be needed to confirm these trends.

### Human Retinal Microvascular Endothelial Cells as a Relevant in Vitro Model to Study TNF-α Induced Cellular Adhesion

**Figure 4.**
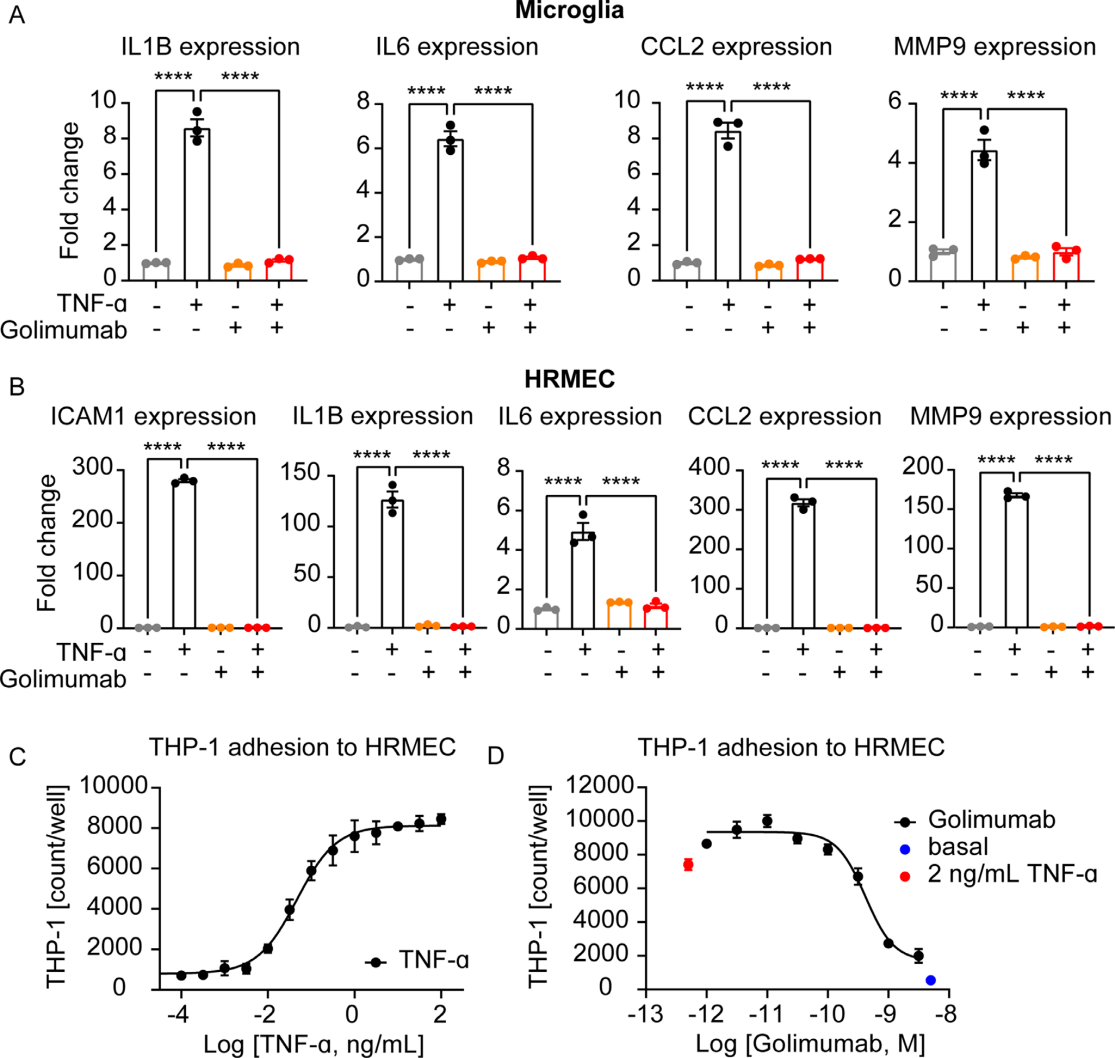
TNF-α induced expression of proinflammatory cytokines and monocyte adhesion to retinal endothelial cells. (A) TNF-α (10 ng/mL) upregulated expression of IL1B, IL6, CCL2, and MMP9 in microglial cells and this upregulation was blocked by treatment with 58.8 nM golimumab (Golimumab ****P < 0.0001, TNF-α: ****P < 0.0001, Interaction: ****P < 0.0001; n = 3). (B) The endothelial cell activation marker ICAM-1 was upregulated upon TNF-α stimulation and downregulated by golimumab treatment in HRMECs (Golimumab ****P < 0.0001, TNF-α: ****P < 0.0001, Interaction: ****P < 0.0001; n = 3). Selected proinflammatory cytokines and chemokines (IL1B, IL6, CCL2 and MMP9) were upregulated by TNF-α and this upregulation was prevented by golimumab treatment (Golimumab ****P < 0.0001, TNF-α: ****P < 0.0001, Interaction: ****P < 0.0001; n = 3). (C) Adhesion of mononuclear THP-1 cells to HRMECs was increased by TNF-α treatment in a dose-dependent manner. (D) Golimumab treatment reduced THP-1 adhesion to HRMECs induced by 2 ng/mL TNF-α in a concentration-dependent manner. Mean ± SEM is shown in all graphs and two-way-ANOVA with Sidak's multiple comparisons test was used for statistical analysis. All in vitro experiments were repeated at least three times in independent experiments.

To assess whether the TNF-α induced cytokine expression and pathologies observed in mice may be relevant for humans, we investigated if our findings can be translated to human in vitro cell culture models. Thus we tested whether TNF-α stimulation activates an immortalized microglial cell line and primary HRMEC and if TNF-α stimulation alters expression of proinflammatory cytokines. Indeed, TNF-α induced expression of *IL1B*, *IL6*, *CCL2* and *MMP9* and this upregulation was prevented in the presence of golimumab (Fig. 4A), suggesting that activation of microglia by TNF-α increases cytokine gene expression. In line with the microglia experiments, stimulation of HRMECs with TNF-α, increased expression of the adhesion molecule *ICAM1*, an effect that was prevented by golimumab. Similarly, TNF-α induced expression of *IL1B*, *IL6*, *CCL2* and *MMP9* in HRMECs, which was prevented by golimumab (Fig. 4B). Our data suggest that both human microglial and retinal endothelial cells can be selectively activated by TNF-α.

To mimic vasculitis observed in AAV-TNF-α treated animals in a functional cellular assay with human cells, we stimulated HRMECs with TNF-α in the presence or absence of golimumab and added fluorescently labeled monocytic THP-1 cells. Interestingly, adhesion of mononuclear THP-1 to HRMECs was increased upon TNF-α stimulation in a concentration dependent manner (Fig. 4C), suggesting that TNF-α also mediates the adhesion of immune cells to human endothelial cells corroborating our in vivo data. Finally, golimumab diminished the TNF-α-mediated THP-1 adhesion to HRMEC cells in a concentration dependent manner (Fig. 4D). Altogether, we present in this study how TNF-α induced retinal inflammation may be studied both in an AAV-TNF-α driven in vivo model and in vitro cell cultures to further understand the function of TNF-α in retinopathies.

## Discussion

In this study, we evaluated whether the AAV-TNF-α induced retinopathy mouse model is susceptible to an anti-inflammatory treatment. AAV-driven expression of approximately 120 pg human TNF-α is stable for at least six weeks,^25^ thus allowing analysis of progressively developing pathologies that resemble disease progression in patients. In this study, we treated AAV-TNF-α–injected eyes with a single IVT injection of the neutralizing TNF-α antibody golimumab to validate our model. To keep golimumab in excess to TNF-α for as long as possible, we injected the maximal dose of 100 µg golimumab in the maximal possible volume of 1 µL per mouse eye. Hence, injection of 100 µg golimumab leads to an approximate ratio of 100,000:1 golimumab to TNF-α (monomer). Ocular concentrations of golimumab are expected to decrease rapidly after IVT injection, as has been shown for other therapeutic antibodies; for example, the IgG bevacizumab has a half-life of eight to 50 hours in the rat eye after IVT injection,^40^^,^^41^ but the half-life of IgGs in the smaller mouse eye should be even shorter. Few studies investigated the pharmacokinetics of IgGs in the murine eye, (e.g., adalimumab and conbercept).^33^^,^^42^ Estimating an ocular half-life of 10 hours for golimumab based on the pharmacokinetics studies of conbercept in mice,^42^ golimumab would be in excess to TNF-α for approximately seven days and should therefore be able to delay the observed phenotypes induced by AAV-TNF-α for seven days. Indeed, the single IVT injection of golimumab was sufficient to reduce retinal thickening, posterior synechiae, and the development of a fibrotic epiretinal membrane; it also partially rescued the impaired ERG response. Of note, AAV-stuffer treatment alone decreased ERG response independent of TNF-α, and this effect may not be affected by golimumab treatment. However, the AAV-stuffer–induced ERG amplitude reduction was much smaller compared to AAV-TNF-α, suggesting that the reduction in AAV-TNF-α injected eyes mostly results from increased TNF-α expression and -to a smaller part- may be caused by the IVT injection per se, the buffer or the injection of any AAV into the eye. We also noticed that the reduced ERG amplitude in AAV-stuffer injected animals is independent of inflammation, since no sign of inflammation were observed based on histological cross-sections at the dose used here (1 ∙ 10^9^ VG/eye). To achieve a full rescue of AAV-TNF-α mediated pathologies, long-term exposure of golimumab may be needed in future studies (e.g., by hydrogels) for sustained release of therapeutic antibodies in the vitreous^43^ or AAV-driven expression of IgG-based biomolecules similar to ADVM-022.^44^ However, care must be taken when designing AAV-based therapeutics, since AAVs itself can induce inflammation in a dose-dependent manner, as seen at the highest AAV dose in the recent INFINITY trial by Adverum. Finally, another reason why golimumab treatment only partially rescued the AAV-TNF-α induced pathologies might be that murine TNF-α is produced by macrophages activated by the exogenous hTNF-α (data not shown), since TNF-α is known to activate macrophages, which in turn, produce more TNF-α.^45^ However, golimumab is not cross-reactive with murine TNF-α^23^ and will therefore only neutralize the AAV-TNF-α induced human TNF-α.

Inflammation is a common hallmark in diverse retinopathies that is not limited to uveitis, but also in AMD and DR and has, thus, come into focus for therapeutic approaches. Uveitis-related pathologies are modeled in rodents by endotoxin- or antigen-induced uveitis^28^^,^^46^ or transgenic animals lacking the *Aire* gene.^47^ Systemic treatment of uveitis with neutralizing antibodies against rodent TNF-α ameliorated pathologies observed in the EAU model, further validating an important role for TNF-α in uveitis.^48^^–^^50^ Similarly, inflammation as indicated by macrophage infiltration is also observed in the laser-induced choroidal neovascularization (CNV), a well-characterized model for wet AMD^51^ and anti-TNF-α treatment reduced lesion area and leakage of CNV.^52^^,^^53^ However, the use of neutralizing TNF-α antibodies in rodent models is limited given the low sequence homology between human and mouse TNF-α (79%), and most human anti-TNF-α therapeutics on the market are not cross-reactive with rodent TNF-α.^23^ In the past, humanized transgenic TNF-α mouse models have been used to overcome these limitations and have proven extremely valuable to understand TNF-α mediated pathologies and to develop novel therapies against arthritis.^23^^,^^54^ Similarly, our AAV-based model is a useful approach to quickly generate humanized animal models to overcome limited conservation of proteins between species. Furthermore, to study the role of TNF-α in diverse organs, transgenic mice overexpressing TNF-α in a tissue-specific manner have been developed, for example in the CNS, lung, or heart.^55^^–^^57^ Although generation of such transgenic animals is time-consuming, expensive, and difficult for non-classical animal models, AAVs can be easily injected into the tissue and species of interest, further highlighting the exceptional potential of AAV-induced disease models to study the biologic function of TNF-α in a tissue-specific manner in the future.

Posterior synechia is the adhesion of the iris to the lens and a common complication of chronic inflammation in uveitis patients. Similarly, our AAV-TNF-α induced uveitis-like mouse model also developed posterior synechiae as seen by a smaller pupil size with irregular borders in vivo, which was confirmed by histological cross-sections. In line with our data, the EAU uveitis mouse model^58^^,^^59^ and a rabbit model for ocular inflammation using intravitreal injection of recombinant TNF-α also developed posterior synechiae,^60^ demonstrating that TNF-α leads to the development of synechiae also in non-rodent species. Interestingly, although the original publication describing the ShH10 capsid that was used in this study to primarily infect Müller glia,^36^ follow-up studies have shown that ShH10 also efficiently transduces the ciliary body.^61^ These data suggest that TNF-α is likely also expressed in the ciliary body, thereby causing inflammation not only in the posterior part of the eye, but also in the anterior part, as seen in our study by posterior synechia and immune cells in the aqueous chamber. Our AAV-TNF-α model is thus also suitable to study the development of posterior synechiae during chronic inflammation.

Fibrosis has been described in diverse retinal diseases and contributes to disease progression^39^ (e.g., subretinal fibrosis secondary to AMD^62^ or fibrotic epiretinal membranes^63^). Interestingly, we also observed fibrosis in our AAV-TNF-α driven model, however, in contrast to AMD, we did not observe subretinal fibrosis, but rather the development of a fibrotic epiretinal membrane-like layer in the inner retina. Fibrotic scarring is also observed in uveitis-models such as the EAU model, but also here fibrosis is rather observed in the outer retinal layers,^64^ probably because of the systemic inflammation induced in the EAU model as opposed to our IVT-based approach. The fibrotic epiretinal membrane in our model likely originates from MMT, because the fibrotic cells were positive for macrophage markers Iba1 and F4/80 but negative for RPE65. Interestingly, epiretinal membranes secondary to uveitis also often contain immune cells, but no RPE cells^65^^,^^66^ in line with our finding in AAV-TNF-α injected mice. To further understand the origin and mechanism of TNF-α-mediated immune cell infiltration, multiparameter flow cytometry will be a valuable tool to further characterize the exact cell types present in our model. MMT has also been described in other rodent models of retinopathies (e.g., the laser-induced CNV model) where F4/80^+^ myofibroblasts have been found in the subretinal space at the site of the laser damage.^38^ Altogether, IVT injection of AAV-TNF-α resembles fibrotic epiretinal membranes observed in human uveitis patients, and it will be interesting to test whether subretinal injection of AAV-TNF-α may lead to subretinal fibrosis in future studies.

We have shown previously that IVT injection of AAV-TNF-α significantly increases expression of the human transgene itself, as well as proinflammatory cytokines and chemokines IL-1β, IL-6, and CCL2/MCP-1^25^ similar to the upregulation of several cytokines and chemokines that have been measured in the vitreous humor of uveitis patients.^67^ In this study, we could confirm a similar trend toward upregulation, but because of a smaller animal number, the changes were only statistically significant for CCL2. Similarly, golimumab treatment did not significantly reduce expression of IL-1β, IL-6, CCL2, and MMP-9 compared to vehicle-treated animals, but a trend was observed. High variation between animals was observed, suggesting that expression of these marker genes should be analyzed in future studies with a larger group size. To verify the observed trends in vivo in a human in vitro system, we studied expression of these markers using primary HRMECs and immortalized microglial cells. However, these microglia are an immortalized cell line that may not completely reflect all characteristics of microglia in vivo; thus they must be used with caution.^68^ Nevertheless, in accordance with our mouse data, we confirmed a strong TNF-α–dependent upregulation of *IL1B*, *IL6*, *CCL2* and *MMP9*, which was fully prevented in the presence of golimumab. Our data are in line with a previous transcriptome analysis that demonstrated upregulation of *IL1B* and *CCL2* after TNF-α stimulation in HRMECs^69^ and microglial activation by TNF-α.^20^ Supporting our findings, serum MMP9^70^ and CCL2^71^ levels have also been described to be downregulated by infliximab therapy in arthritic patients. Interestingly, MMP9 has also been linked to experimental CNV^72^ and peripheral blood mononuclear cells from neovascular AMD patients produce higher levels of CCL2.^73^ Altogether, our data fit very well with the previously described important role of cytokine secretion by activated microglia in the disease progression of retinopathies.^10^^,^^74^

Finally, we have shown in this study that AAV-TNF-α induced vasculitis can be translated to a human cell-culture based assay using a monocyte-to-retinal-endothelium adhesion assay. This approach demonstrates the relevance of the observed phenotypes in our AAV-TNF-α induced mouse model for human diseases, allows high-throughput approaches and further minimizes the use of animal models in accordance with animal welfare guidelines. TNF-α-induced monocyte-to-endothelial-cell adhesion assays have a long history and are frequently used, especially in the context of atherosclerosis research. Human umbilical vein endothelial cells or human aortic endothelial cells are the major endothelial cell types used in the literature for TNF-α induced adhesion assays,^75^^–^^77^ but only few studies have previously used retinal vascular cells to extend this approach to retinal diseases. For example, a flow-chamber-based adhesion assay was established to measure TNF-α–induced increased monocyte adhesion to retinal vascular endothelial cells^22^^,^^78^ indicating that retinal vascular cells may be used in cell adhesion assays to study retinopathies. Here, we present an easy-to-use TNF-α–dependent monocyte adhesion assay using fluorescently labeled THP-1 cells and HRMECs that do not require elaborate equipment such as a flow-chamber system.

Altogether, we presented evidence that intravitreal AAV-TNF-α injection induces both morphological but also functional deficits in the mouse retina, which is partially rescued by a single dose of neutralizing TNF-α antibody. Our data suggest that our model may be used in the future to further study the function of TNF-α in retinal inflammation and is susceptible to anti-inflammatory treatments. Furthermore, this study is a proof of concept that AAV-driven expression of a retinopathy-associated gene can be modulated by a single IVT injection of a neutralizing antibody, highlighting the great potential of AAV-induced animal models.

## Supplementary Material

Supplement 1
