# Supplementary material for: Characterization and Validation of In Vitro and In Vivo Models to Investigate TNF-α-Induced Inflammation in Retinal Diseases
Source: Transl Vis Sci Technol. 2022 May 17;11(5):18. doi: 10.1167/tvst.11.5.18 (PMC9123507; doi:10.1167/tvst.11.5.18)
Supplement: Supplement 1 [file tvst-11-5-18_s001.pdf]

Supplemental Figures

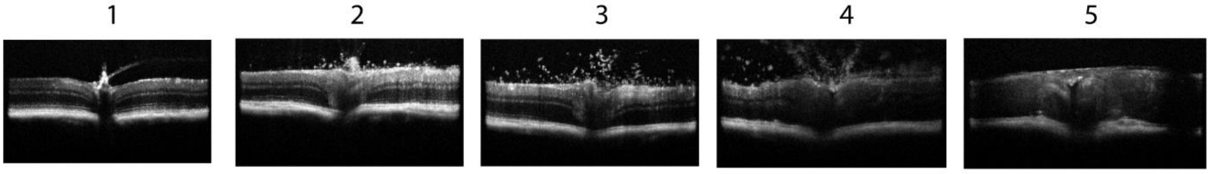

Supplemental Figure 1: Examples of inflammation grading based on OCT images. Scores from 1 (healthy) to 5 (severely affected) were given to OCT images based on number of cellular infiltrates in vitreous and (dis-)organization of retinal layers.

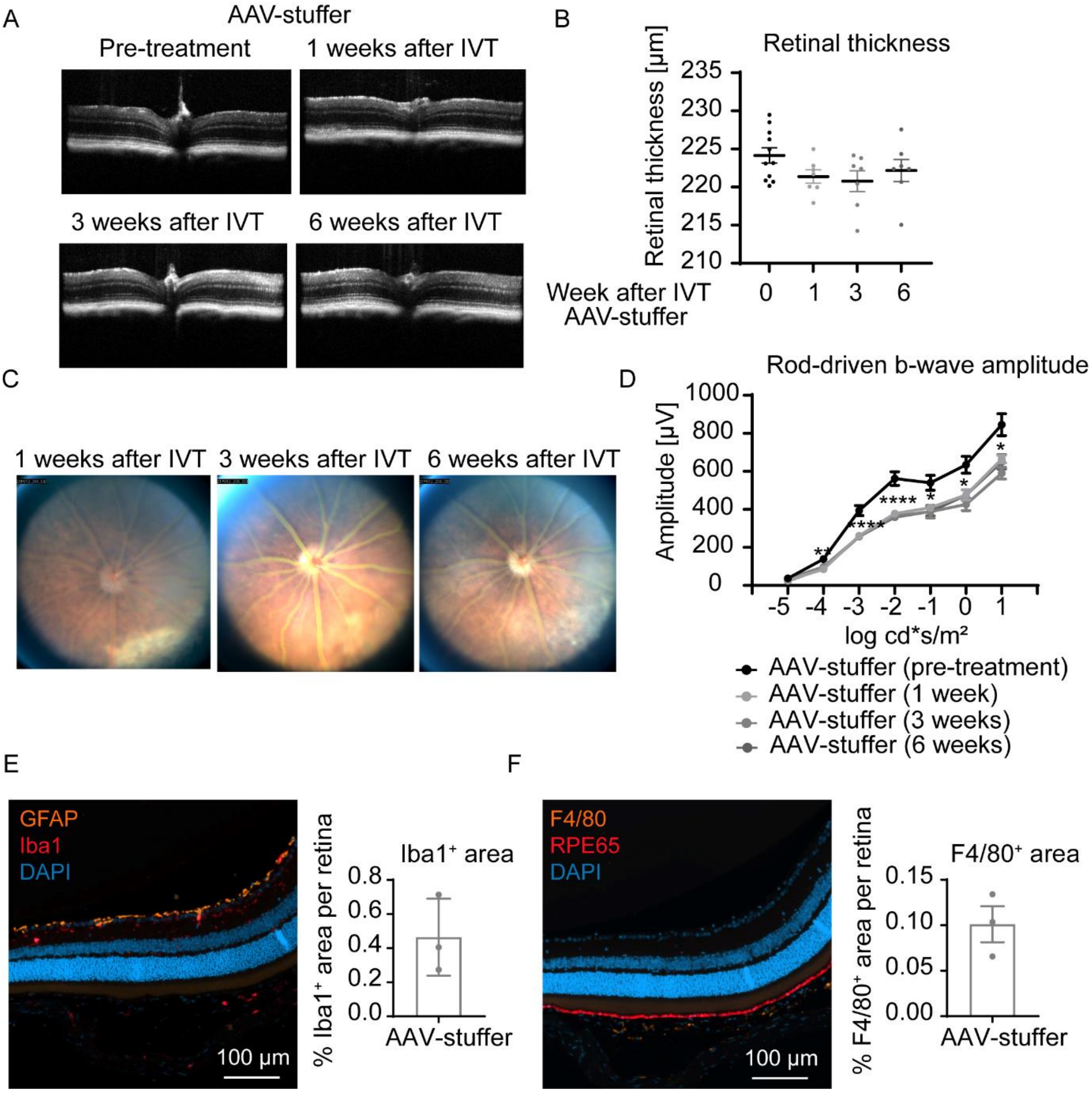

Supplemental Figure 2: Retinal thickness is not altered, but ERG response is reduced by AAV-stuffer injection. (A) OCT scans of the same animal before and 1, 3 and 6 weeks after injection of AAV-stuffer. (B) Retinal thickness is not significantly altered after AAV-stuffer injection compared to the pre-treatment control in the same animals *(n.s.; Mixed-effects analysis with Dunnett's post-hoc test in comparison to pre-treatment value, n=7-12) (C) Fundus pictures of AAV-stuffer injected eyes appear mostly normal apart from few bright spots. (D) Rod-driven b-wave amplitude is reduced 1, 3 and 6 weeks after injection of AAV-stuffer compared to the pre-treatment control (Mixed-effects analysis with Dunnett's post-hoc test in comparison to pre-treatment value of each flash intensity: ****p<0.0001; **p<0.01, *p<0.05, n=7.12).* Note that the same animals were measured before IVT, 1, 3 and 6 weeks after IVT of AAV-stuffer. (E) GFAP staining (orange) of AAV-stuffer injected eyes showed that GFAP was restricted to the astrocyte layer and no Müller glia activation was observed. Only

few microglia/macrophages (Iba1, red) that were restricted to the plexiform layers were found in AAV-stuffer treated eyes. (F) Activated macrophages (F4/80, orange) were mostly absent from AAV-stuffer injected eyes and the RPE monolayer appeared normal (RPE65, red). Nuclei were stained with DAPI (blue). Histological cross-sections of AAV-stuffer injected eyes were collected from an independent mouse cohort. Mean ± SEM is shown in all graphs

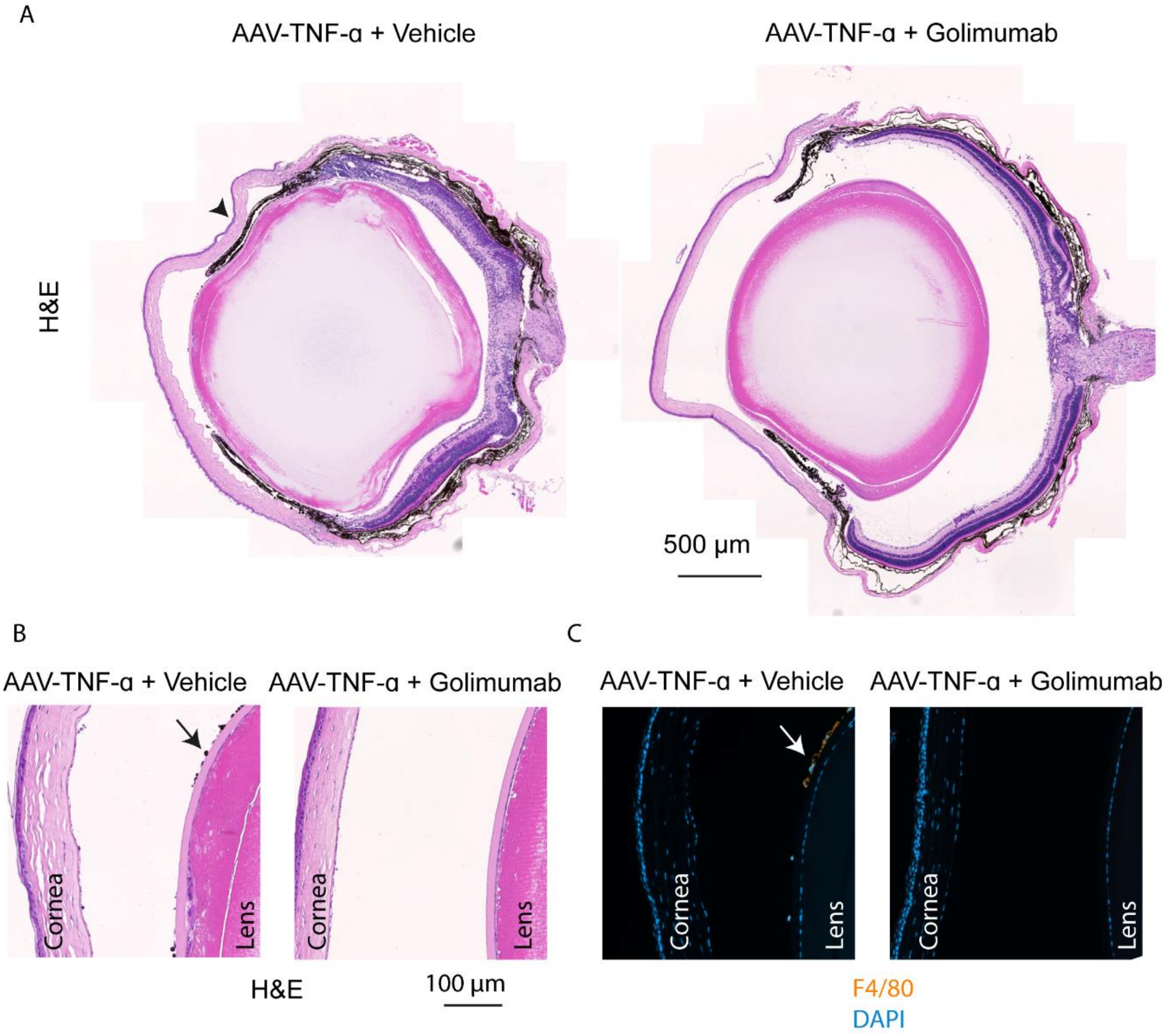

Supplemental Figure 3: H&E stained cross-sections of the eye showed overall improved retinal morphology in golimumab treated eyes. (A) Golimumab treated eyes had clearly distinguishable retinal layers in contrast to vehicle treated eyes. Representative pictures show posterior synechia (black arrowhead) in vehicle, but not golimumab treated mice. (B) Zoom in of panel A. Cellular infiltrates were observed in the anterior chamber of AAV-TNF-α injected eyes, but not in the golimumab treated eyes. (C) Cellular infiltrates in the anterior chamber were positive for the activated macrophage marker F4/80 (orange). Nuclei are shown in blue.

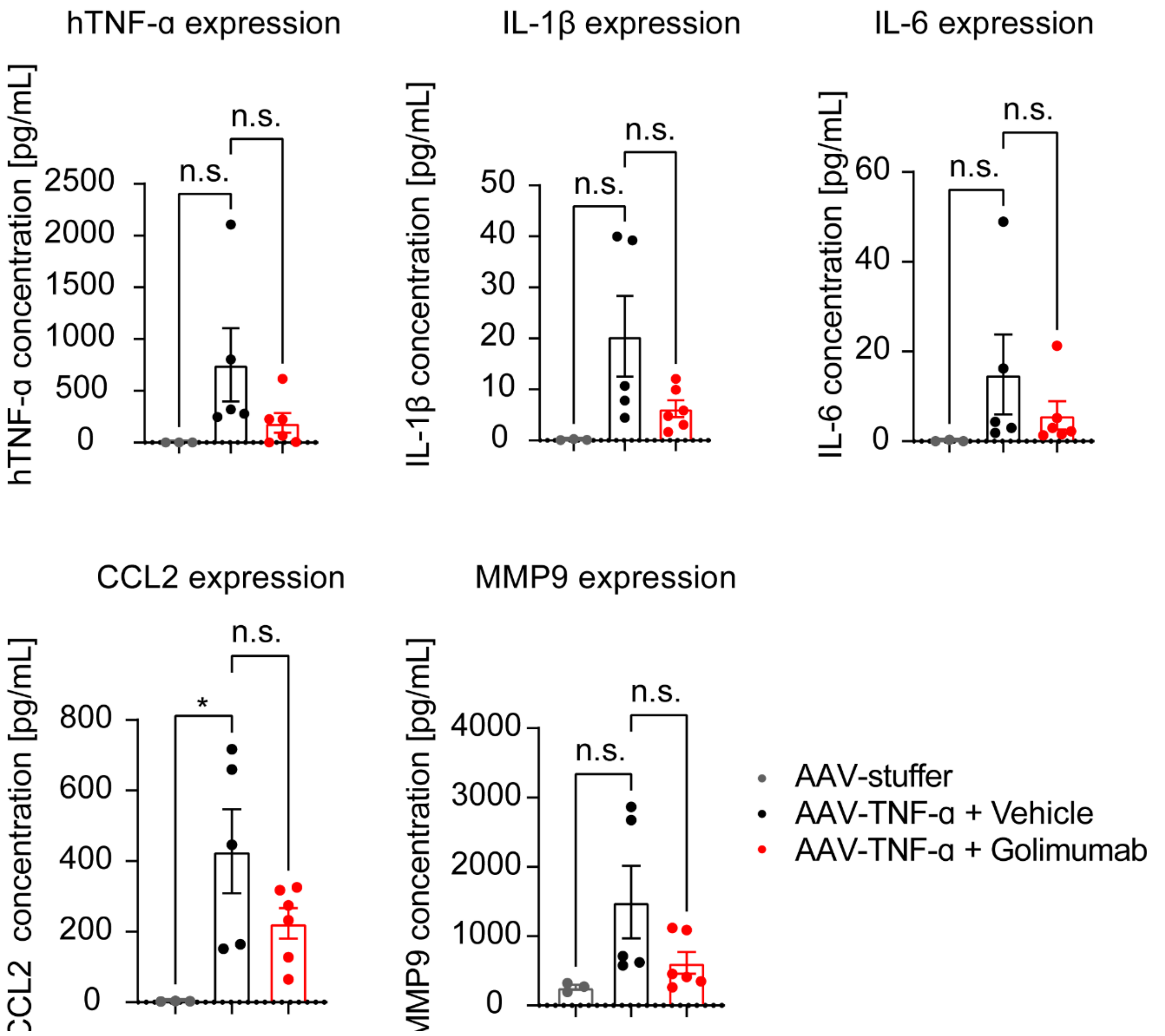

Supplemental Figure 4: Expression analysis of human TNF- α and murine IL-1β, IL-6, CCL2 and MMP9 in mouse eye lysates after IVT injection of AAV-TNF-α and golimumab. Human TNF-α and mouse IL-1β, IL-6, CCL2 and MMP9 proteins were slightly upregulated in AAV-TNF-α injected eyes, but only the upregulation of CCL2 was statistically significant *(CCL2: *$p<0.05$; 1-way-ANOVA with Tukey post-hoc test; mean ± SEM is shown in all graphs, n=3-6)*. Golimumab treatment only mildly reduced the TNF-α induced upregulation of the selected cytokines, but none of the changes was significant.
